# Supplementary material for: Low-Dose Empagliflozin Improves Systolic Heart Function after Myocardial Infarction in Rats: Regulation of MMP9, NHE1, and SERCA2a
Source: Int J Mol Sci. 2021 May 21;22(11):5437. doi: 10.3390/ijms22115437 (PMC8196699; doi:10.3390/ijms22115437)
Supplement: Supplementary file 1 [file ijms-22-05437-s001.zip › ijms-1211563-supplementary.pdf]

Supplementary Material

Expanded Materials and Methods

Ultrasonic measurements

Transabdominal ultrasound measurements of aortic diameters were performed preoperatively, and on day 7 postoperatively, using the Vevo 3100 (VisualSonics Inc., Toronto, Canada) equipped with a 25 MHz single-crystal transducer with a focal length of 15 mm, a frame rate of 40 Hz and the maximum field of view of 2D imaging 21x21mm. Rats were anesthetized with 1.5-2 % isoflurane with oxygen and placed on a heated platform in the supine position. M-mode tracings were recorded from short- axis view of the left ventricle (LV) at the level of the papillary muscles with two-dimensional image guidance through the anterior and posterior walls. Left ventricular internal dimensions were measured through the largest diameter of the left ventricle, both at the end of diastole (LVIDd) and systole (LVIDs). Ejection fraction (EF) and fractional shortening (FS) were calculated from the linear measurements of LVIDd and LVIDs. Pulsed wave Doppler spectra of mitral inflow was recorded from the apical four chamber view to evaluate diastolic parameters: E- wave- early passive ventricular filling velocity, A- wave- late atrial ventricular filling velocity, deceleration time (EDT) and E/A- ratio. All measurements were performed in a blinded manner: The information about the animals was coded and masked from the experimenter. All data were evaluated using the VevoLab software (Version 2.2.0, VisualSonics Inc., Toronto, Canada).

Table S1. Antibodies.

**Target Antigen**	**Source**	**Catalog #**	**Working Concentration**	**Lot #**
MMP9	Abcam, Hiddenhausen, Germany	ab38898	1:1000	GR3204084-9
MMP2	Abcam, Hiddenhausen, Germany	ab110186	1:500	GR82901-1
TIMP1	Santa Cruz Biotechnology Inc., Germany	sc-5538	1:500	H0311
TGF-β1	Santa Cruz Biotechnology Inc., Germany	sc-146	1:500	H0311
MLKL	*Merck* Millipore, Darmstadt, Germany	MABC604	1:1000	2677923
NCX1	Marly, Swant, Switzserland	R3F1	1:500	mr05
NBC1	Cohesion Bioscience, London, UK	CQA1189	1:500	AL1F04A
NHE1	Abbiotec, San Diego, USA	251167	1:500	
Serca 2a	Dianova, Hamburg, Germany		1:500	
GAPDH	Abcam, Hiddenhausen, Germany	MAB374	1:10000	3189695
Anti-mouse polyclonal goat	Dako Denmark A/S, Glostrup	P0260	1:2000	20014498
Anti-Rabbit polyclonal goat	Dako Denmark A/S, Glostrup	P0217	1:2000	00086784
ABC-Kit	Vectastain ABC, Vector Laboratories	PK-4000		
AEC-Kit	AEC, Vector Laboratories, Burlingame, USA	SK-4200		
